# The Naturally Evolved *EPSPS* From Goosegrass Confers High Glyphosate Resistance to Rice

**DOI:** 10.3389/fpls.2021.756116

**Published:** 2021-10-29

**Authors:** Chao Ouyang, Wei Liu, Silan Chen, Huimin Zhao, Xinyan Chen, Xiongxia Jin, Xinpeng Li, Yongzhong Wu, Xiang Zeng, Peijin Huang, Xiuying He, Baoguang An

**Affiliations:** ^1^Hainan Bolian Rice Gene Technology Co., Ltd., Haikou, China; ^2^Guangdong Provincial Key Laboratory of New Technology in Rice Breeding, Guangzhou, China

**Keywords:** glyphosate, *EPSPS*, goosegrass, rice, plant transformation

## Abstract

Glyphosate-resistant crops developed by the *CP4-EPSPS* gene from *Agrobacterium* have been planted on a massive scale globally, which benefits from the high efficiency and broad spectrum of glyphosate in weed control. Some glyphosate-resistant (GR) genes from microbes have been reported, which might raise biosafety concerns. Most of them were obtained through a hygromycin-*HPT* transformation system. Here we reported the plant source with 5-enolpyruvylshikimate-3-phosphate synthase (*EPSPS*) gene from goosegrass endowed rice with high resistance to glyphosate. The integrations and inheritability of the transgenes in the rice genome were investigated within two generations. The *EiEPSPS* transgenic plants displayed similar growth and development to wild type under no glyphosate selection pressure but better reproductive performance under lower glyphosate selection pressure. Furthermore, we reconstructed a binary vector pCEiEPSPS and established the whole stage glyphosate selection using the vector. The Glyphosate-pCEiEPSPS selection system showed a significantly higher transformation efficiency compared with the hygromycin-*HPT* transformation system. Our results provided a promising alternative gene resource to the development of GR plants and also extended the plant transformation toolbox.

## Introduction

Glyphosate is the most widely used herbicide in global agriculture since the 1970s due to its high efficiency on non-selective and broad-spectrum inhibition of annual and perennial weeds (Duke, 2018). Glyphosate disrupts the shikimate pathway by competitively binding to the 5-enolpyruvylshikimate-3-phosphate synthase (EPSPS) with phosphoenolpyruvate (PEP) (Marzabadi et al., 1996; McDowell et al., 1996). This endows glyphosate low toxicity risks to humans because the shikimate pathway is found only in microbes and plants, and not in animals (Herrmann and Weaver, 1999). Glyphosate also benefits farmers and the environment with its low cost and easy degradation. Therefore, breeders welcome glyphosate-resistant (GR) crops to suppress various kinds of weeds without affecting the fitness of crop plants. There are two strategies to create GR crops (Guo et al., 2015). One is to eliminate the toxic residues of glyphosate through acetylation, oxidative cleavage, and/or reduced translocation, such as glyphosate N-acetyltransferase (GAT) (Castle et al., 2004; Siehl et al., 2005) and glyphosate oxidoreductase (GOX) (Pedotti et al., 2009). This is called the non-target site resistance strategy. Another one is the target site resistance strategy. It overproduces the EPSPS, which could be achieved by introducing a tolerant *EPSPS* gene and/or elevated EPSPS activity *in vivo*.

The EPSPSs from different organisms were divided into two classes previously, according to their intrinsic glyphosate sensitivity (Pollegioni et al., 2011; Yi et al., 2015). Class I enzymes, found in all plants and many Gram-negative bacteria, were naturally sensitive to glyphosate. However, belonging to class I, the *IvEPSPS* from *Isoptericola variabilis* (Yi et al., 2015; Cui et al., 2016) and the *aroA*_*J. Sp*_ from *Janibacter* sp. (Yi et al., 2016) rendered rice resistant to glyphosate. Class II enzymes, found only in microbes, were naturally glyphosate-tolerant, which could be used directly to create GR plants. The most famous class II EPSPS was from the *Agrobacterium* sp. strain CP4, which was used in the first commercial GR soybean (Funke et al., 2006) and was also introduced to rice (Chhapekar et al., 2014). The EPSPS from *Staphylococcus aureus* was also insensitive to glyphosate (Priestman et al., 2005). The *G2-EPSPS* from *Pseudomonas fluorescens* and the *G6-EPSPS* from *P. putida* were developed to breed GR cotton, soybean, maize (Guo et al., 2015; Liu et al., 2015; Zhang et al., 2016) and rice (Zhao et al., 2011), respectively.

Apart from these EPSPSs from microbes, GR weeds with mutations in EPSPSs generated from the intense glyphosate selection pressure have provided an alternative approach for crop improvements for decades (Chandrasekhar et al., 2014; Sammons and Gaines, 2014; Heap and Duke, 2018). Globally, the number of weeds species evolving to GR climbs to 57 (Heap, 2021) after the first GR weed was reported in the Rigid Ryegrass in Australia (Pratley et al., 1996; Powles et al., 1998). The single amino acid substitution of P106S in the plant EPSPS was identified in the spontaneously occurring GR goosegrass (*Eleusine indica*) until 2002 (Baerson et al., 2002), which corresponded to the substitution of P101S in the EPSPS from GR *Salmonella typhimurium* (Comai et al., 1983; Stalker et al., 1985). Then several other substitutions of P106 in EPSPS (P106T, P106A, or P106L) were found in GR goosegrass and Rigid Ryegrass (Ng et al., 2004; Yu et al., 2007; Kaundun et al., 2011). Nevertheless, these single-codon EPSPS mutations showed a lower affinity for the substrate PEP and endowed plants with low-level glyphosate resistance. The double-codon EPSPS mutations [T102I + P106S (TIPS)] using site−directed mutagenesis obtained high affinity for PEP and tolerance to glyphosate in *Escherichia coli* (Funke et al., 2009). The maize EPSPS was mutated into TIPS EPSPS through this method, which has been successfully used to produce the first commercial transgenic glyphosate-tolerant maize (GA21) (Spencer et al., 2000). Through DNA shuffling, the mutated GR EPSPSs from *Malus domestica* (T101A and A187T) and *Vitis vinifera* were verified in Arabidopsis and rice (Tian et al., 2013, 2015). The *Os-mEPSPS* (P173S) produced by site-directed mutagenesis was identified to have a tolerance to glyphosate in rice (Chandrasekhar et al., 2014). Additionally, powerful genome editing tools have also been applied to generate endogenous GR EPSPS in plants (Li et al., 2016; Hummel et al., 2018; Sammons et al., 2018; Arndell et al., 2019; Sedeek et al., 2019). However, only the *CP4-EPSPS* gene mentioned above has been commercialized successfully. Biosafety concerns about the heterologous expression of the microbial EPSPSs in plants urged the development of novel gene resources. Recently, a naturally evolved TIPS-EPSPS mutation was confirmed to be highly resistant to the glyphosate in goosegrass, which exerted a 180-fold higher resistance than the wild type (WT) and 32-fold higher than the previously known P106S mutants (Yu et al., 2015). However, whether the *TIPS-EPSPS* mutated goosegrass gene could be applied to GR breeding remains unknown.

In the present study, the *TIPS-EPSPS* from goosegrass (designated as *TIPS-EiEPSPS*) was heterologously expressed in rice, conferring high resistance to glyphosate. The integrations of the transgenes in the rice genome were detected using PCR and quantitative PCR (qPCR) analyses. The inheritability and agronomic performances were investigated with T_1_ generation. Furthermore, we reconstructed a binary vector pCEiEPSPS based on pCAMBIA1300 (Hajdukiewicz et al., 1994) and provided the whole stage glyphosate selection based on the pCEiEPSPS vector, including calli selection, regeneration, rooting, and seed germination, which was more comprehensive and efficient than the previous studies. Our study speaks volumes about *TIPS-EiEPSPS* providing a promising alternative gene resource to the development of GR plants.

## Results

### Codon Usage Optimization of *TIPS-EiEPSPS* and Construction of a Novel Plant Binary Transformation Vector pCEiEPSPS

**FIGURE 1:**
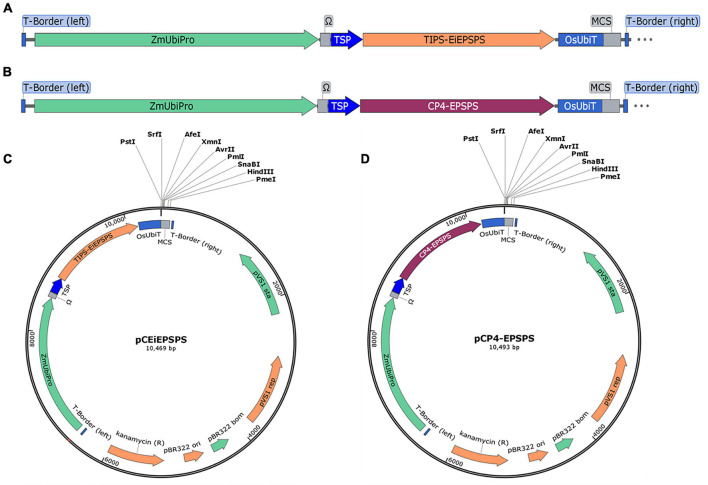
Schematic representation of the plant binary expression vector. **(A,B)** The expression cassette of *TIPS*-*EiEPSPS* and *CP4-EPSPS*, respectively. **(C,D)** The plant binary expression vectors pCEiEPSPS and pCP4-EPSPS containing the *EiEPSPS* expression cassette and *CP4-EPSPS* expression cassette, respectively, with the same multiple cloning sites (MCS). ZmUbipro, maize ubiquitin promoter; Ω, enhancer; TSP, chloroplast target peptide from tobacco; OsUbiT, rice ubiquitin terminator; MCS, multiple cloning sites.

There were a few glyphosate tolerance genes reported previously, however, most of them were introduced into plants using the hygromycin-*hygromycin phosphotransferase* (*Hpt*) or other selection systems (Tian et al., 2013; Chandrasekhar et al., 2014; Liang et al., 2017; Achary et al., 2020). Therefore, we aimed to set up a selection system to replace the *Hpt* expression cassette with a plant-sourced GR expression cassette. Then the mutated *TIPS-EPSPS* from the goosegrass and the chloroplast target peptide from tobacco were synthesized after being codon usage optimized using OptimumGene (GenSript, Piscataway, NJ, United States), which was placed under the control of the maize Ubiquitin promoter (ZmUbipro) with an enhancer (Ω) (Figure 1A). The *TIPS-EiEPSPS* expression cassette was constructed into the binary vector pCAMBIA1300 to replace the *Hpt* expression cassette using the *Pme*I and *Sac*II, which was designated as the binary vector pCEiEPSPS (Figure 1C). The class II EPSPS from the *Agrobacterium* sp. strain CP4 (*CP4-EPSPS*) was also cloned in the same way as *TIPS-EiEPSPS* for the comparison of their glyphosate tolerance, which was designated as the binary vector pCP4-EPSPS (Figures 1B,D). These two expression cassettes were introduced into rice by an *Agrobacterium*-mediated Transformation.

### Whole Stage Glyphosate Selection Based on the pCEPSPS Vector

**FIGURE 2:**
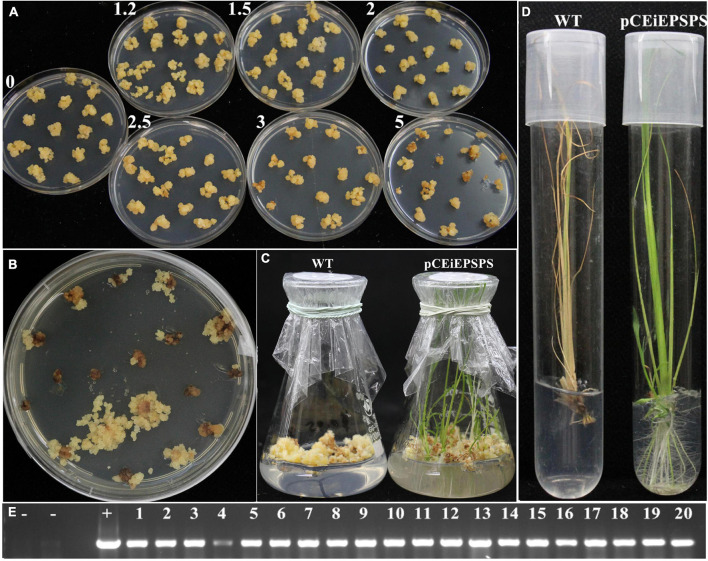
Transformation using whole-step selection by glyphosate-based on pCEiEPSPS vector. **(A)** Glyphosate tolerance tests for the ZH11 callus. The numbers represent different glyphosate concentrations (mM) in the subculture medium. **(B)** Selection of positive callus on the selection medium containing 3 mM glyphosate after about 40 days of infection. **(C)** Thirty-five-day-old regenerated shoots under the 0.05 mM glyphosate pressure. Wild type (WT), ZH11 callus; pCEiEPSPS, positive callus from panel **(B)**. **(D)** Two-week-old rooting plantlets under the 0.05 mM glyphosate pressure. WT, ZH11 regenerating plantlets; pCEiEPSPS, positively regenerated plantlets from panel **(C)**. **(E)** The positive transgenic events were determined by PCR using the *EiEPSPS* specific primers (UEU-F1 and UEU-R1). Lane -, -, H_2_O, and ZH11 genomic DNA used as the negative control, respectively; Lane +, the vector pCEiEPSPS used as the positive control; Lane 1–20, independent pCEiEPSPS transgenic events.

To construct the glyphosate selection system, we performed whole stage selection tests including selection, regeneration, rooting, and seed germination. Generally, the glyphosate concentration in the selection medium was a magnitude higher than that in the regeneration, rooting, or seed germination media (Supplementary Figure 1). During the selection stage, glyphosate could repress the growth of non-transgenic calli at the concentration of 2.5 mM. Lower than that, there would be some new non-transgenic callus arising, which will make it difficult to differentiate the transgenic positive callus from the non-transgenic callus and affect the regeneration of green shoots (Figure 2A, Supplementary Figure 3, and Supplementary Table 1). In our research, the glyphosate concentration at the selection medium could be as high as 5 mM without significantly affecting the positive calli selection efficiency (Table 1). During the regeneration stage, glyphosate could inhibit the differentiation of non-transgenic calli into the shoots at a very low concentration of 0.05 mM. In contrast, the transgenic calli differentiated into the shoots under 0.15 mM of glyphosate pressure without significantly affecting the regeneration efficiency, which was inhibited when the glyphosate pressure was above 1 mM. The transgenic shoots still emerged under 3 mM glyphosate pressure (Supplementary Figure 1A and Supplementary Table 2). The situation of the rooting stage was similar to that of the regeneration stage, except that the transgenic shoots could tolerate higher glyphosate (4 mM) with slight growth inhibition (Supplementary Figure 1B and Supplementary Table 3). A similar trend was observed in the seed germination stage, where the selection pressure in the rooting medium distinguished the transgenic and non-transgenic seeds (Supplementary Figure 1C and Supplementary Table 4). However, the selection pressure in the hydroponic culture was slightly higher than that in the rooting medium (Supplementary Figure 2). It was noted that there was no difference in the selection pressure between the two major subspecies, *indica* and *japonica* (Supplementary Figures 1C, 2).

**TABLE 1:** *Agrobacterium*-mediated transformation efficiency using the binary vector pCEiEPSPS and pCP4-EPSPS.

Vector	Selective agent	Selection efficiency (%)	Regeneration efficiency (%)	Rooting efficiency (%)	Positive transgene events by PCR (%)	Final transformation efficiency (%)
pCEiEPSPS	3 mM glyphosate	55.18 ± 7.21a	92.61 ± 4.89a	82.87 ± 3.98a	100 ± 0a	42.07 ± 4.12a
	5 mM glyphosate	58.51 ± 14.37a	92.66 ± 2.25a	94.02 ± 4.24b	100 ± 0a	51.24 ± 14.16a

pCP4-EPSPS	3 mM glyphosate	60.63 ± 14.04a	81.87 ± 2.06b	93.21 ± 6.41b	100 ± 0a	45.386.59a
	5 mM glyphosate	59.33 ± 11.33a	82.85 ± 8.08b	93.92 ± 6.68b	100 ± 0a	46.31 ± 0.65a

pC1300	50 mg/L hygromycin	47.65 ± 2.73a	89.11 ± 3.47a	65.95 ± 15.68c	86.67 ± 2.36b	27.79 ± 6b

*Data are the means ± SDs, different letters represent the significant difference by t-test, p < 0.05.*

### The Glyphosate-pCEiEPSPS Selection System Displayed High Transformation Efficiency

After the measurement of the glyphosate selection pressure during the tissue culture, an *Agrobacterium*-mediated transformation was performed to create GR rice using the binary vector pCEiEPSPS and pCP4-EPSPS, separately. Both of these binary vectors showed a high transformation efficiency (Figures 2B–D, Table 1, and Supplementary Table 5). We noted that the transformation rate using the glyphosate-*EPSPSs* selection system was much higher (42–51%) than that using the hygromycin-*Hpt* selection system (27.79%), indicating the high efficiency of the glyphosate-*EPSPSs* selection system.

To further identify whether the candidate *EPSPS* genes were introduced into the rice genome, PCR was performed using the genomic DNA samples extracted from the seedlings during acclimatization. The results showed that all the pCEiEPSPS rooted seedlings were transgenic plants (Figure 2E) and there were no false-positive transgenic events, which might be owed to the whole-stage selection.

The transgene copy number was next analyzed by qPCR in the T_0_ generation using the genomic DNA samples above. The single-copy endogenous gene, *sucrose phosphate synthase* (*SPS*), was used as the internal reference gene. The transgene copy number was also predicted by the glyphosate resistance segregation of the T_1_ progeny. The ratios of the single-copy transgene events in both tests were 25–36.4% for the pCEiEPSPS and pCP4-EPSPS binary vector, respectively (Table 2), offering plenty of options to pick out the stable transgenic events. Statistically, the average transgene copy numbers for the events generated from the pCEiEPSPS and pCP4-EPSPS binary vectors were 2.3 and 2.1 copies per genome, respectively (Table 2). Overall, the Glyphosate-pCEiEPSPS selection system displayed a high transformation efficiency and extremely low false-positive events with acceptable transgene copy numbers.

**TABLE 2:** Transgene copy number of the binary vector pCEiEPSPS and pCP4-EPSPS detected by segregation and quantitative PCR (qPCR).

Binary vector	Transgenic line	Total progeny	Resistant progeny	Susceptible progeny	Segregation ratio	X^2^c	*P*-value	Predicted copy number by segregation	Predicted copy number by qPCR
pCEiEPSPS	L1	36	8	28	3:1	50.70	*p* < 0.05	Complicated	1
	L2	40	32	8	3:1	0.30	*p* > 0.05	1	1
	L3	32	24	8	3:1	0.04	*p* > 0.05	1	3
	L4	28	14	14	3:1	8.05	*p* < 0.05	Complicated	2
	L5	52	47	5	15:1	0.51	*p* > 0.05	2	3
	L6	36	34	2	15:1	0.03	*p* > 0.05	2	2
	L7	42	31	11	3:1	0.00	*p* > 0.05	1	5
	L8	44	32	12	3:1	0.03	*p* > 0.05	1	1
	L9	38	27	11	3:1	0.14	*p* > 0.05	1	1
	L10	25	14	11	3:1	3.85	*p* < 0.05	Complicated	1
	L11	18	18	0	15:1	0.37	*p* > 0.05	≥2	1
	L12	25	7	18	3:1	27.00	*p* < 0.05	Complicated	2
	L13	36	35	1	15:1	0.27	*p* > 0.05	≥2	3
	L14	30	24	6	3:1	0.18	*p* > 0.05	1	1
	L15	45	44	1	15:1	0.65	*p* > 0.05	≥2	7
	L16	48	36	12	3:1	0.03	*p* > 0.05	1	2
Average copy number									2.3 ± 1.64

pCP4-EPSPS	L1	32	22	10	3:1	0.38	*p* > 0.05	1	1
	L2	45	33	15	3:1	0.94	*p* > 0.05	1	1
	L5	40	30	10	3:1	0.03	*p* > 0.05	1	1
	L7	27	25	2	15:1	0.02	*p* > 0.05	2	3
	L8	36	36	0	15:1	1.45	*p* > 0.05	≥2	3
	L9	54	52	2	15:1	0.24	*p* > 0.05	≥2	4
	L10	20	2	18	3:1	41.67	*p* < 0.05	Complicated	2
	L11	45	42	3	15:1	0.04	*p* > 0.05	2	2
	L12	25	24	1	15:1	0.00	*p* > 0.05	≥2	3
	L13	26	21	5	3:1	0.21	*p* > 0.05	1	2
	L14	27	21	6	3:1	0.01	*p* > 0.05	1	1
Average copy number									2.1 ± 1.00

### *EiEPSPS* Confers High Glyphosate Resistance in Rice

**FIGURE 3:**
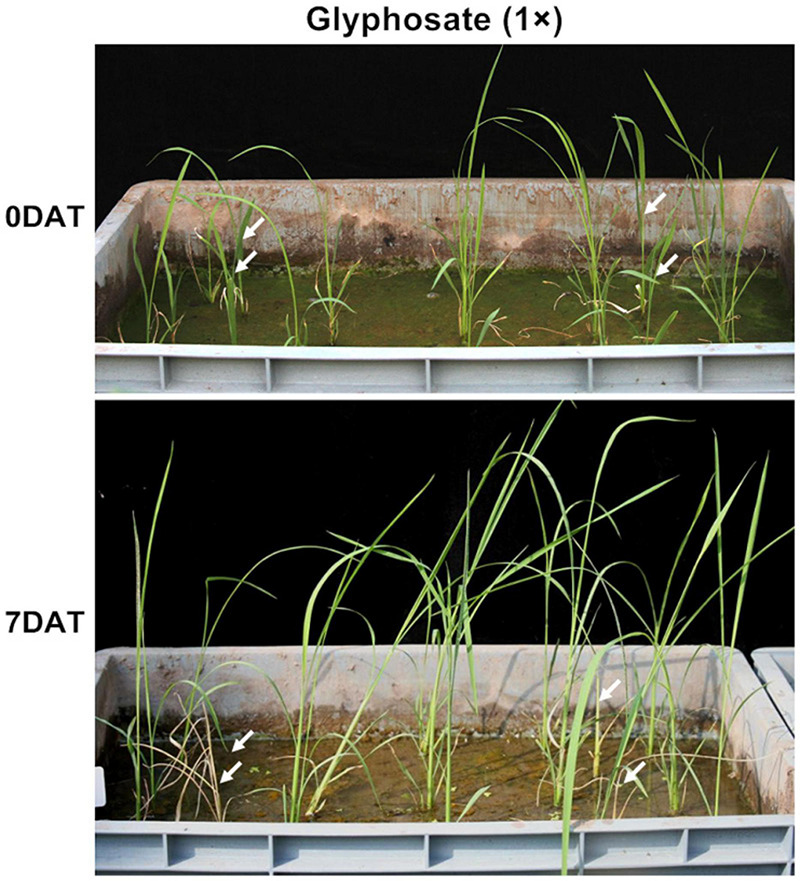
The glyphosate-resistance level test of the pCEiEPSPS T_0_ transgenic plants. Fifteen pCEiEPSPS T_0_ transgenic plantlets and four WT plantlets (white arrows) were planted into the soil per pot. The phenotypes were recorded before [0 days after treatment (DAT)] and 7 (7DAT) days after spraying 0.1125 g a.e./m^2^ (1×) glyphosate. No WT plantlet survived from glyphosate while most of the pCEiEPSPS T_0_ transgenic plantlets grew normally.

The GR level of the T_0_ transgenic seedlings was determined at the 3–4-leaf stage after being transplanted into the soil by foliar spraying. All the *TIPS-EiEPSPS* and *CP4-EPSPS* transgenic plants displayed normal growth while the WT plants died under the treatment with 0.1125 g a.e./m^2^ (1×) glyphosate for 7 days (Figure 3 and Table 3). Based on the high survival rate after treatment with low concentration, we treated the transgenic plants of pCEiEPSPS and pCP4-EPSPS with 2×, 5×, and 10× glyphosate to further compare the resistant levels of *TIPS-EiEPSPS* and *CP4-EPSPS*. These findings displayed that more than 80% of the *TIPS-EiEPSPS* transgenic plants and more than 86.67% of the *CP4-EPSPS* transgenic plants grew well after being treated with 2× glyphosate (including high and moderate resistance). Most of the *TIPS-EiEPSPS* transgenic plants were in good conditions, under the 5× and 10× glyphosate treatment. However, most of the *CP4-EPSPS* transgenic plants showed a low resistance level to 5× and 10× glyphosate (Table 3), indicating that *TIPS-EiEPSPS* could contribute to the better resistance ability to glyphosate than *CP4-EPSPS*.

**TABLE 3:** Glyphosate resistance analysis of the T_0_ transgenic lines.

Binary vector	Glyphosate dosage (g a.e./m^2^)	Total number of transgenic lines	Number of HR lines	HR ratio (%)	Number of MR lines	MR ratio (%)	Number of LR lines	LR ratio (%)	Number of NR lines	NR ratio (%)
ZH11	0.1125 (1×)	30	0	0	0	0	0	0	30	100.00

pCEiEPSPS	0.1125 (1×)	69	63	91.30	4	5.80	2	2.90	0	0.00
	0.225 (2×)	10	6	60.00	2	20.00	1	10.00	1	10.00
	0.5625 (5×)	9	8	88.89	0	0.00	1	11.11	0	0.00
	1.125 (10×)	10	7	70.00	2	20.00	1	10.00	0	0.00

pCP4-EPSPS	0.1125 (1×)	35	31	88.57	2	5.71	2	5.71	0	0.00
	0.225 (2×)	15	5	33.33	8	53.33	2	13.33	0	0.00
	0.5625 (5×)	13	1	7.69	0	0.00	11	84.62	1	7.69
	1.125 (10×)	13	2	15.38	3	23.08	4	30.77	4	30.77

*HR, High Resistant, plants with no injury at all after being sprayed by glyphosate; MR, Moderate Resistant, plants with some injury spots after being sprayed by glyphosate but would recover growth in a week; LR, Low Resistant, plants with severe injury but still alive; NR, None Resistant.*

### Field Test of the *EiEPSPS* Transgenic Lines With Glyphosate Treatment

**FIGURE 4:**
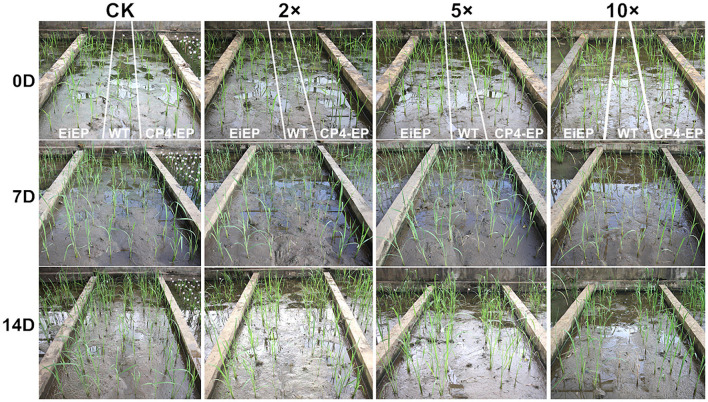
Field test of the glyphosate resistance in the pCEiEPSPS and pCP4-EPSPS T_1_ transgenic plants. The seeds from the pCEiEPSPS and pCP4-EPSPS T_1_ transgenic plants were germinated under glyphosate selection and the seedlings were transplanted in the field with three lines and the WT plant were planted with two lines between them (showed between the white lines). After about 2 weeks of being transplanted in the field, the plants were treated with 0, 0.225 (2×), 0.5625 (5×), or 1.125 (10×) g a.e./m^2^. The phenotypes were recorded at 7 and 14 days after being sprayed. All the WT plants died at 14DAT while the pCEiEPSPS and pCP4-EPSPS T_1_ transgenic plants grew normally. CK, plants without glyphosate treatment; EiEP, pCEiEPSPS T_1_ transgenic lines; CP4-EP, pCP4-EPSPS T_1_ transgenic lines.

To evaluate the agricultural potential of the *TIPS-EiEPSPS* transgenic lines under glyphosate application, the T_1_ transgenic lines with only one transferred (T)-DNA copy were treated with different dosages of glyphosate at the vegetative stage in the field. And the agronomic traits were examined at the reproductive stage. Most of the agronomic traits were similar between the WT and the *TIPS-EiEPSPS* or *CP4-EPSPS* transgenic lines in the control treatment except that the panicle numbers were slightly higher in the *CP4-EPSPS* transgenic lines L1, L2 (Table 4). The WT plants could not survive after any dosage of glyphosate application (Figure 4). Compared with the WT without glyphosate application, the *TIPS-EiEPSPS* or *CP4-EPSPS* transgenic lines under all the glyphosate applications showed no significant differences in the panicle length, seed set rate, and 1,000-grains weight. However, the panicle number of most of the transgenic lines significantly increased under the glyphosate treatment compared with the control treatment, especially under the 0.225 g a.e./m^2^ (2×) glyphosate application and the grain number per panicle was also increased in the *TIPS-EiEPSPS* transgenic lines L8 and L9. These findings implied that a low level of glyphosate stress might be helpful to reproductive growth and development. The increase of these two traits consequently contributed to the increase of the yield per plant (Table 4). Under 10× glyphosate treatment, although the panicle number of the *TIPS-EiEPSPS* transgenic lines significantly increased, the yield of all the transgenic lines was similar to the WT. This finding indicated that the application of high concentrations of glyphosate in the tilling stage would not affect the yield production of the transgenic lines. The situation of 5× glyphosate application was similar to that of the 10× glyphosate. These results suggest that *pCEiEPSPS* has a great potential in developing GR plants. Notably, the *TIPS-EiEPSPS* transgenic lines displayed similar agronomic traits with the *CP4-EPSPS* transgenic lines except the slightly higher plant heights under the 0.225 g a.e./m^2^ (2×) glyphosate application. The *TIPS-EiEPSPS* transgenic lines are of value and should be further investigated of field performances at a large scale.

**TABLE 4:** Glyphosate resistance analysis of the T_1_ transgenic lines in the field.

Binary vector	Transgenic line	Glyphosate dosage (g a.e./m^2^)	Plant height	No. of panicles	Panicle length	1,000-grain weight	Grain No. per panicles	Filled grains per panicle	Seed-set rate (%)	Yield per plant
ZH11		CK	80.52 ± 4.73a	5.67 ± 1.44a	20.04 ± 1.77a	23.8	95.49 ± 18.2a	53.28 ± 18.1a	55.18 ± 13.09a	8.93 ± 3.33a

pCEiEPSPS	L2	CK	81.41 ± 5.45a	6.17 ± 0.68a	19.44 ± 1.79a	23.8	90.77 ± 26.1a	55.22 ± 20.2a	66.06 ± 9.81a	8.93 ± 2.25a
	L8		83.60 ± 4.70a	6.97 ± 1.83a	18.50 ± 2.30a	23.0	88.00 ± 25.4a	48.66 ± 10.8a	53.16 ± 13.00a	7.93 ± 2.30a
	L9		79.75 ± 4.01a	7.33 ± 2.21a	19.33 ± 2.03a	22.5	99.16 ± 18.0a	46.83 ± 17.5a	46.44 ± 11.75a	7.30 ± 1.02a
	L14		80.75 ± 1.99a	5.83 ± 2.03a	20.35 ± 1.47a	21.2	100.29 ± 21.8a	61.28 ± 12.3a	56.32 ± 8.97a	7.85 ± 2.58a
pCP4-EPSPS	L1		84.00 ± 1.70a	7.67 ± 1.24b	17.37 ± 1.87*c*	23.9	87.68 ± 28.1a	52.74 ± 19.0a	60.15 ± 9.08a	6.32 ± 1.81a
	L2		82.16 ± 6.02a	8.50 ± 1.89b	20.05 ± 2.09a	24.4	97.93 ± 22.1a	66.11 ± 17.4a	61.46 ± 10.59a	9.95 ± 2.61a
	L5		84.50 ± 2.91a	6.33 ± 0.74a	20.61 ± 1.47a	23.7	104.68 ± 21.8a	66.70 ± 15.1a	63.78 ± 9.12a	8.67 ± 2.17a

pCEiEPSPS	L2	0.225 (2×)	79.50 ± 2.30a	8.50 ± 2.21b	19.58 ± 1.73a	24.7	92.10 ± 18.9a	57.11 ± 15.3a	62.32 ± 10.43a	12.71 ± 2.03b
	L8		87.25 ± 3.61b	7.25 ± 1.29b	20.35 ± 1.66a	22.0	129.23 ± 26.2b	80.50 ± 20.7b	62.32 ± 11.25a	10.63 ± 1.21a
	L9		85.58 ± 4.43b	9.00 ± 1.15b	20.27 ± 1.09a	24.5	119.57 ± 19.4b	71.32 ± 15.1a	59.96 ± 10.62a	9.53 ± 2.03a
	L14		86.75 ± 2.86b	8.67 ± 2.28b	20.92 ± 1.46a	23.4	113.40 ± 25.7a	68.24 ± 19.0a	60.47 ± 11.76a	10.31 ± 2.39a
pCP4-EPSPS	L1		78.25 ± 3.99a	9.33 ± 1.97b	18.45 ± 4.91a	22.4	94.60 ± 24.6a	58.28 ± 17.6a	62.61 ± 11.33a	10.55 ± 2.63a
	L2		81.75 ± 4.12a	8.83 ± 3.48b	20.55 ± 1.93a	21.5	104.03 ± 23.6a	70.08 ± 20.7a	67.37 ± 11.06a	10.78 ± 3.40a
	L5		82.75 ± 2.59a	6.00 ± 1.15a	20.61 ± 2.04a	24.2	98.40 ± 25.8a	66.08 ± 19.8a	66.47 ± 12.28a	10.05 ± 2.09a

pCEiEPSPS	L2	0.56 ± 25 (5×)	76.41 ± 1.94a	7.83 ± 2.33b	18.91 ± 1.66a	24.3	95.94 ± 19.5a	59.40 ± 10.3a	60.24 ± 7.27a	6.62 ± 1.89a
	L8		86.50 ± 4.61b	6.50 ± 1.70a	19.48 ± 1.59a	22.8	101.32 ± 41.7a	70.46 ± 19.9a	62.70 ± 9.95a	7.80 ± 4.00a
	L9		76.91 ± 4.01a	6.67 ± 1.69a	19.54 ± 0.91a	21.9	109.67 ± 15.3a	65.72 ± 17.6a	59.24 ± 10.50a	6.20 ± 1.96a
	L14		71.81 ± 3.70*c*	6.30 ± 0.81a	20.74 ± 1.44a	22.5	102.20 ± 19.5a	61.84 ± 16.4a	60.50 ± 11.14a	5.65 ± 2.34a
pCP4-EPSPS	L1		72.75 ± 2.62*c*	7.67 ± 2.28b	17.77 ± 1.17*c*	21.5	91.02 ± 17.5a	56.15 ± 11.6a	62.54 ± 11.14a	6.82 ± 2.19a
	L2		77.66 ± 2.19a	8.33 ± 2.05b	18.68 ± 1.02a	21.9	100.35 ± 21.7a	55.85 ± 15.9a	55.61 ± 10.84a	8.08 ± 1.58a
	L5		78.91 ± 5.71a	5.83 ± 0.68a	20.52 ± 0.84a	24.4	94.71 ± 15.6a	60.46 ± 10.9a	64.53 ± 10.27a	8.77 ± 1.70a

pCEiEPSPS	L2	1.125 (10×)	76.41 ± 1.94a	9.17 ± 1.34b	19.44 ± 1.71a	24.4	104.26 ± 17.2a	61.94 ± 16.9a	62.75 ± 17.43a	9.05 ± 3.14a
	L8		87.25 ± 3.35b	9.50 ± 3.59b	20.34 ± 2.36a	20.6	123.00 ± 33.1b	72.11 ± 24.5a	58.78 ± 13.13a	8.58 ± 2.90a
	L9		82.16 ± 4.81a	7.20 ± 1.32a	21.18 ± 1.48a	22.0	112.09 ± 15.6a	71.21 ± 12.5a	64.45 ± 12.83a	7.08 ± 3.21a
	L14		83.50 ± 3.02a	7.33 ± 0.74b	21.09 ± 1.46a	20.8	115.50 ± 27.6a	65.53 ± 14.7a	58.61 ± 13.58a	8.08 ± 2.69a
pCP4-EPSPS	L1		79.66 ± 1.81a	7.00 ± 1.15a	18.82 ± 1.48a	20.7	109.22 ± 18.5a	65.15 ± 17.1a	60.40 ± 13.86a	7.93 ± 2.99a
	L2		82.66 ± 4.00a	9.50 ± 2.29b	19.32 ± 1.41a	22.5	117.13 ± 23.1a	64.00 ± 20.3a	57.37 ± 10.47a	9.75 ± 3.57a
	L5		84.16 ± 3.19a	7.00 ± 1.63a	20.87 ± 2.08a	22.4	113.97 ± 32.0a	69.43 ± 26.1a	61.07 ± 11.59a	8.55 ± 3.89a

*Data are means ± SDs, different letters represent the significant difference by t-test, p < 0.05.*

## Discussion

There have been numerous reports about the application of different GR EPSPSs in creating GR plants in recent years after the first commercial CP4-EPSPS transgenic crops were created (Funke et al., 2006). Most of them were cloned from microbes, which might raise security concerns. We provided the naturally evolved GR *TIPS-EPSPS* gene from goosegrass, a kind of plant, which would be beneficial for the positive promotion of transgenic products.

Among the GR genes and plants mentioned above, most of them were obtained through hygromycin-*HPT* transformation system or other selection methods and few were created through transformation by glyphosate selection uniquely. Cui et al. (2016) reported that the addition of 200 mg L^–1^ of glyphosate (about 1.2 mM) in the selection medium would help in generating the *IvEPSPS* positive transgenic rice events with about 99% transformation efficiency. Zhao et al. (2011) reported that the addition of 2 mM of glyphosate in the selection medium and 0.1 mM in the rooting medium gave rise to the *G6-EPSPS* positive transgenic rice events, but they did not present the transformation efficiency. The *G2-EPSPS* was transformed into maize *via* a three-round selection with gradually increasing glyphosate concentration (Liu et al., 2015). All of these three studies only used the GR *EPSPS* expression cassette to create the GR transgenic plants. Additionally, the *GR79-EPSPS* from *P. stutzeri* and *GAT* were successfully co-expressed in cotton by a selection with 100 mg L^–1^ glyphosate (about 0.6 mM), but there was an *NPTII* expression cassette retained between the T-DNA borders (Liang et al., 2017). A new study reported that the overexpression of the *TIPS-OsEPSPS* conferred high resistance to glyphosate in rice while our research was being performed. Nonetheless, they used the Hygromycin-*HPT* selection system to generate transgenic rice plants (Achary et al., 2020). The *NPTII* expression cassette in the GR cotton study and the *HPT* expression cassette in most of the other studies barely contribute to the safety assessment of genetically modified organisms (GMOs) or the further commercial application of the GMOs. Hence, it would be more appropriate to create the GR plants only with GR genes and the glyphosate selection system.

There might be differences in the glyphosate selection concentration among different crops. The glyphosate concentration used in our study also differed from the previous reports in rice. For the transformation of the *IvEPSPS*, 1.2 mM glyphosate was used (Cui et al., 2016), and 2 mM glyphosate was used in the transformation of the *G6-EPSPS* (Zhao et al., 2011), respectively. We also tested these two concentrations in the selection medium. Surprisingly, the results showed that the positive calli from the 1.2 mM glyphosate selection would regenerate more albino shoots (Supplementary Figure 3). There were no significant differences in the appearance of the calli, which was not mentioned in the previous study. Two millimoles glyphosate in the selection medium would reduce the ratio of the albino shoots which were not fully eliminated (data not shown). The differences might be caused by the different resistance levels among the EPSPSs or among the time duration and methods of the selection stage. Based on our results, we recommend a little higher glyphosate concentration (2.5–5 mM) in the selection medium, which would significantly inhibit the albino shoots during the regeneration. Our results were consistent with a new report in *indica* rice (Hu et al., 2021). Combining the selection pressure in the regeneration, rooting, and seed germination stage, we provided a stable glyphosate selection system with high efficiency. which was much higher than that using the hygromycin-*Hpt* selection system. This might result from the resistant EPSPSs which contribute to the PEP assimilation besides the native EPSPS, which was conducive to plants. While the hygromycin-*Hpt* selection system only acted as an inhibitor and played no positive role in plants.

The *TIPS-EiEPSPS* T_0_ transgenic plants displayed high resistance to glyphosate after the whole-stage selection. When the T_1_ generation was treated with different dosages of glyphosate at the early vegetative stage, the transgenic progenies showed high resistance. A low dosage of glyphosate application (2×) might promote reproductive growth, whereas a high dosage of glyphosate application (5× or 10×) would inhibit the growth and reduce the yield per plant. There was no significant yield increase under any dosage of glyphosate application, which was consistent with the results from the *IvEPSPS* transgenic plants (Cui et al., 2016) and varied from the results from the *TIPS-OsEPSPS* (Achary et al., 2020). This might have resulted from the differences in the sample size. A larger-scale test should be performed to investigate the contribution of *TIPS-EiEPSPS* to rice production. In-depth research including the resistance level at different development stages and in other crops such as maize or wheat also needs to be implemented. Anyhow, *TIPS-EiEPSPS* could be, at least, comparable with *CP4-EPSPS* in the role of developing GR plants.

Overall, by cloning the *TIPS-EPSPS* of goosegrass and reconstructing the binary vector pCEiEPSPS, we established the whole stage glyphosate selection system. It possessed a high transformation efficiency and extremely low false-positive transgenic events, which consequently endowed rice with high resistance to glyphosate. Our research provided an alternative GR gene from plant genomes, which was at least comparable to the resistant level of *CP4-EPSPS* and would extend the plant transformation toolbox.

## Materials and Methods

### Plant Materials and Growth Conditions

Rice plants [*O. sativa* L. ssp. *japonica* “Zhonghua 11” (ZH11 in short)] were grown in a greenhouse with a 16/8 h light/dark photoperiod at 30/20°C or under field conditions at Haikou, Hainan province, China during the summer in 2019–2020.

### Codon Usage Optimization and Construction of Plant Binary Transformation Vectors

The nucleotide sequence of the mutated *TIPS-EiEPSPS* was obtained from goosegrass (Yu et al., 2015), led by the tobacco chloroplast target peptide. Then the coding domain was optimized according to the rice codon usage using OptimumGene (GenSript, Piscataway, NJ, United States), which was placed under the control of *ZmUbipro* with the Ω enhancer (Gallie, 2002) and a rice Ubiquitin terminator (*OsUbiT*). For the convenience of constructing the novel binary vector, a whole expression cassette with an upstream additional 319 bp sequence between the *Sac*II and the 35S polyA of the pCAMBIA1300 vector and a downstream of multiple cloning site sequence was synthesized, which was ligated into the linearized pCAMBIA1300 vector (digested by *Sac*II and *Pme*I) using a Lightening Cloning Kit (Gene-Foci, Beijing, China). The primers were 1300-UEU-F (5′-AGCCGATTTT GAAACCGCGGTGATCACAGGCAGCAACGC-3′) and 1300-UEU-R (5′-TCCTGTCAAACACTGATAGTTTAAACTGAAG GCGGGAAACGACAATC-3′). The resulting novel plant binary vector was designated as the pCEiEPSPS (GenBank NO. MZ351488). After being verified by sequencing, the plasmid was transferred into the *Agrobacterium tumefaciens* strain EHA105 for the rice transformation. The binary vector pCP4-EPSPS was constructed and used the same way as pCEiEPSPS.

### Critical Concentration Tests and *Agrobacterium*-Mediated Transformation

The glyphosate concentrations in the media at different stages of the tissue culture were determined before the transformation. For the critical concentration test of induction, subculture, and selection, the embryogenic calli were placed on the media supplemented with 0, 1.2, 2, 2.5, 3, and 5 mM glyphosate, respectively, in the dark for 5–6 weeks to observe whether new calli were emerging or not. For the critical concentration test of regeneration, positive selection calli were placed on a regeneration medium supplemented with 0, 0.05, 0.15, 1, and 2 mM glyphosate in the light for 4–5 weeks. For the critical concentration test of rooting, the regenerated seedlings were placed on a rooting medium supplemented with 0, 0.05, 0.1, 0.15, 2, and 4 mM glyphosate in the light for 2–3 weeks. For the critical concentration test of seed germination, the surface-sterilized rice seeds were germinated in a 1/2MS medium supplemented with 0, 0.01, 0.05, 0.2, 0.4, and 0.6 mM glyphosate, respectively, in the light for 2 weeks.

After the determinations above, the *Agrobacterium*-mediated transformation was performed as previously described (Hiei et al., 1994) with modifications. After being infected, the embryogenic calli were placed on a selection medium supplemented with 3–5 mM glyphosate instead of hygromycin for 5–6 weeks. The positive calli were regenerated under 0.05 mM glyphosate selection pressure for 4–5 weeks. And the regenerated seedlings were placed on a rooting medium supplemented with 0.05 mM glyphosate. The transformation experiments were performed at least three times. The efficiency of the selection was calculated as the number of the resistant calli divided by the number of the inoculated calli. The efficiency of the regeneration was calculated as the number of the calli with regenerated shoots divided by the number of the inoculated calli on the regeneration medium. The efficiency of the rooting was calculated as the number of rooted plantlets divided by the number of shoots on the rooting medium. The final transformation efficiency of the rooting was calculated as the number of positive transgene events divided by the number of initial calli. A statistical analysis was conducted using a *t-*test.

Genomic DNA was extracted from young leaves using the cetyltrimethylammonium bromide (CTAB) method. The positive transgenic seedlings were determined by PCR using the primers UEU-F1 (5′-ACCGTGACAGGACCACAGAG-3′) and UEU-R1 (5′-CAAAAGGGTATAGCAGAAGCAA-3′) and were transplanted into the soil in the greenhouse after acclimatization.

### Glyphosate Tolerance Assay in Transgenic Plants

The T_0_ transgenic plants were grown in a greenhouse and then foliar sprayed with glyphosate doses of 0, 0.1125, 0.225, 0.5625, and 1.125 g a.e./m^2^, respectively, using a small handheld sprayer to kill the non-resistant or low-resistant events. The resistant levels of the transgenic lines were divided into four levels by phenotype. After being sprayed by glyphosate, the transgenic lines with no injury at all were categorized to the high resistance level, the transgenic lines that showed some injury spots after being sprayed by glyphosate but would recover growth in a week were categorized to the moderate resistance level, the transgenic lines with severe injuries but still lived were categorized to the low resistance level; and the dead or dying transgenic lines belonged to the no resistance level or sensitive level. The transgenic T_1_ seeds and wild type were sowed and planted in the greenhouse or field, which were foliar sprayed at the four- to five-leaf stage with glyphosate doses of 0, 0.225, 0.5625, and 1.125 g a.e./m^2^ using an agro-atomizer to determine the GR level. The recommended glyphosate (Zhejiang Jinfanda Biochemical Co., Ltd.) dose for plant production is 1,125 g a.e./ha (i.e., 1,125 g a.e./m^2^) according to the manual of the manufacturer, and this concentration was termed as 1×. The application volume was at the rate of 100 mL/m^2^ with different concentrations of glyphosate. The phenotypes were observed at 7 and 14 days after spraying.

### Copy Number Analysis by Quantitative PCR

The copy number of the T-DNA inserted into the rice genome was determined by qPCR using the PIKOREAL 96 (Thermo Fisher Scientific, Waltham, MA, United States) real-Time PCR system. Sixteen pCEiEPSPS transgenic lines and 11 pCP4-EPSPS transgenic lines were randomly selected for the copy number analysis. Quantitative PCR was performed in a 10 μL reaction mixture containing 5 μL of 2× SYBR Mixture, 0.25 mM of each primer, and 40 ng of the genomic DNA. The amplification procedure was 94°C for 7 min followed by 40 cycles of 94°C for 15 s, 55°C for 15 s, 72°C for 15 s (fluorescence detection), and 60°C for 30 s. The melt-curve analysis was performed to confirm that only one product has been amplified. The procedure was a 60–95°C cycle of being increased by 0.2°C and kept for 1 s each cycle. The copy number was estimated using the 2^–ΔΔ*Ct*^ method (Livak and Schmittgen, 2001). The single-copy endogenous gene *SPS* of rice was used as the internal reference gene (Yang et al., 2005). The qPCR primers for *EiEPSPS*, *CP4-EPSPS*, and *SPS* are EiEPSPSqPCR-F (5′-ATGCACACGCAAGACGTTCC-3′) and EiEPSPSqPCR-R (5′-CAGCAGCAGCTCTATGGCTGAG-3′), CP4-EPSPSqPCR-F (5′-AGCGTGGTCACTGCACAGAT-3′) and CP4-EPSPSqPCR-R (5′-GATGGCTGATGGACTTGTCG-3′) and SPSqPCR-F (5′-TTGCGCCTGAACGGATAT-3′) and SPSqPCR-R (5′-CGG TTGATCTTTTCGGGATG-3′), respectively.

### Agronomic Performances of T_1_ Transgenic Lines

At maturity, the important agronomic traits, including plant height, tiller number, panicles per plant, panicle length, filled grains per panicle, 1,000-grain weight, and yield per plant, were measured using at least six randomly selected plants from each transgenic line. The plant height and the number of panicles were measured at the ripening stage. The rest of the characteristics were measured at the end of the plant cycle. The data were collected at one time in Haikou in 2020. Statistical analysis was conducted using a *t-*test between the transgenic lines and the WT.

## Data Availability Statement

The datasets presented in this study can be found in online repositories. The names of the repository/repositories and accession number(s) can be found below: https://www.ncbi.nlm.nih.gov/genbank/, MZ351488.

## Author Contributions

BA and CO conceived and designed the experiments. CO, WL, SC, HZ, XC, XJ, and XZ performed the experiments. BA, CO, and HZ analyzed the data and wrote the manuscript. BA, YW, and XH revised the manuscript. XL and PH contributed to the interpretation of the results. All authors read, revised, and approved the final manuscript.

## Conflict of Interest

CO, SC, HZ, XC, XJ, XL, YW, XZ, PH, and BA were employed by the Hainan Bolian Rice Gene Technology Co., Ltd. The remaining authors declare that the research was conducted in the absence of any commercial or financial relationships that could be construed as a potential conflict of interest.

## Publisher’s Note

All claims expressed in this article are solely those of the authors and do not necessarily represent those of their affiliated organizations, or those of the publisher, the editors and the reviewers. Any product that may be evaluated in this article, or claim that may be made by its manufacturer, is not guaranteed or endorsed by the publisher.
