# Supplementary material for: The Naturally Evolved EPSPS From Goosegrass Confers High Glyphosate Resistance to Rice
Source: Front Plant Sci. 2021 Oct 29;12:756116. doi: 10.3389/fpls.2021.756116 (PMC8586540; doi:10.3389/fpls.2021.756116)
Supplement: Supplementary file 1 [file Data_Sheet_1.PDF]

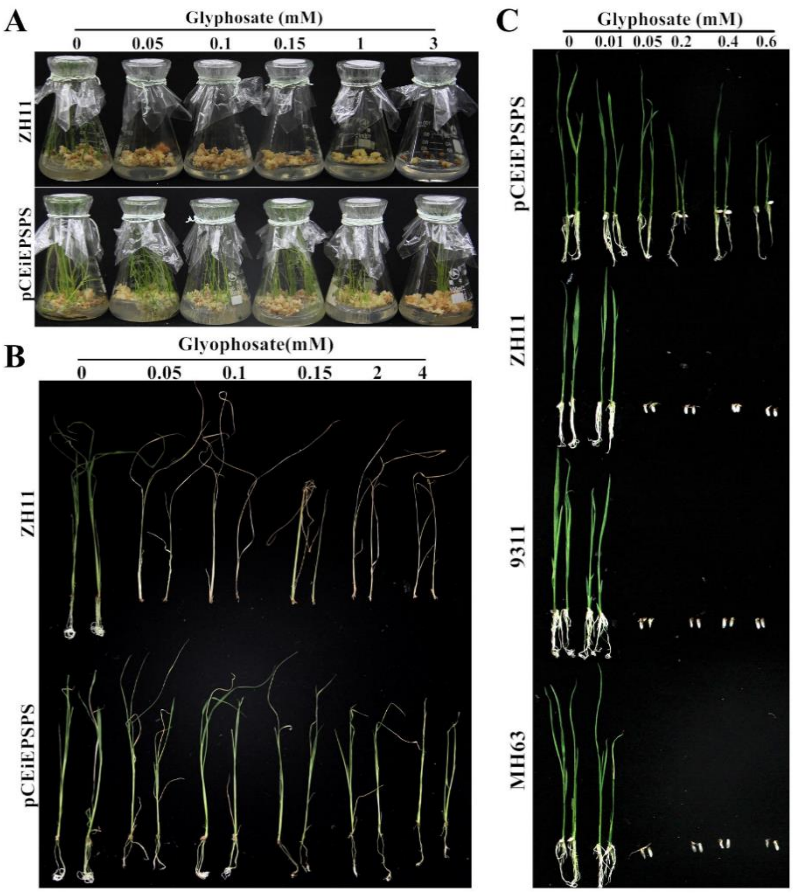

Figure S1. Whole stage selection tests by glyphosate during transformation based on pCEiEPSPS vector.

A, Glyphosate tolerance tests for regeneration. The regeneration of non-transgenic calli (ZH11) were fully inhibited by glyphosate at the concentration of 0.05 mM, while the transgenic calli (pCEiEPSPS) were not significantly affected by 1 mM glyphosate. The phenotypes were recorded after regeneration for 1 month. B, Glyphosate tolerance tests for the rooting of regenerated shoots. The non-transgenic shoots (ZH11) were all killed by glyphosate at a concentration of 0.05 mM or above, while the transgenic shoots (pCEiEPSPS) were survived by 4 mM glyphosate but higher concentration of glyphosate would suppress the growth. The phenotypes were recorded after rooting for 2 weeks. C, Glyphosate tolerance tests for the rooting of seeds, which were similar to the rooting of regenerated shoots. 9311 and MH63 are the *indica* rice cv. 93-11 and Minghui63 for short, respectively. The numbers represent different glyphosate concentrations (mM) in media.

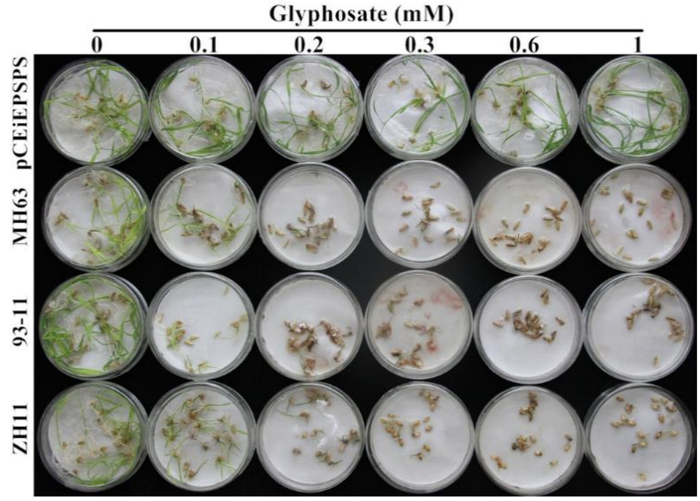

Figure S2. Hydroponic culture selection tests by glyphosate based on pCEiEPSPS vector.
The non-transgenic seeds of *japonica* rice cv. ZH11 and *indica* rice cv. 93-11 and Minghui63 (MH63) were significantly suppressed by glyphosate at the concentration of 0.2 mM and were all killed by glyphosate at the concentration of 0.6 mM or above, while the transgenic seeds (pCEiEPSPS) were not affected by 1 mM glyphosate. The phenotypes were recorded after rooting for 10 days. The numbers represent different glyphosate concentrations (mM) in hydroponic culture.

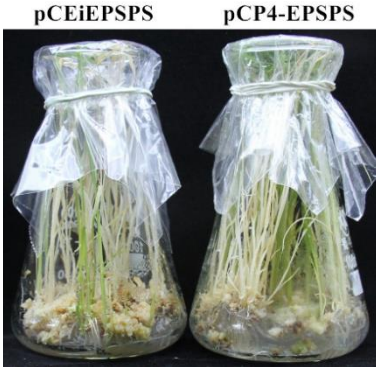

Figure S3. The regenerated albino shoots using the positive calli from selection media containing 1.2 mM glyphosate.
Both of the pCEiEPSPS and pCP4-EPSPS transgenic calli from selection media containing low concentration of glyphosate regenerated lots of albino shoots.

Supplementary Table 1. Effects of different concentration glyphosate on non-transgenic calli in selection.

Medium+glyphosate (mM/L)	Non-transgenic calli		
	No.of calli inoculated	No.of newly grown calli	Ratio (%)
N6S	45	43	95.56
N6S+glyphosate 1.2	45	35	77.78
N6S+glyphosate 2	45	30	66.66
N6S+glyphosate 2.5	45	0	0
N6S+glyphosate 3	45	0	0
N6S+glyphosate 5	45	0	0

Supplementary Table 2. Effects of different concentration glyphosate on regeneration.

Medium+glyphosate (mM/L)	Non-transgenic calli			Transgenic calli		
	No.of calli for regeneration	No.of calli regenerating shoots	Ratio (%)	No.of calli for regeneration	No.of calli regenerating shoots	Ratio (%)
MSRe	45	43	95.56	45	41	91.11
MSRe+glyphosate 0.05	45	0	0	45	42	93.33
MSRe+glyphosate 0.15	45	0	0	45	41	91.11
MSRe+glyphosate 1	45	0	0	45	40	88.89
MSRe+glyphosate 2	45	0	0	45	40	88.89

Supplementary Table 3. Effects of different concentration glyphosate on rooting.

Medium+glyphosate (mM/L)	Non-transgenic shoots			Transgenic shoots		
	No.of shoots for rooting	No.of rooted shoots	Ratio (%)	No.of shoots for rooting	No.of rooted shoots	Ratio (%)
1/2MSR	10	10	100	10	10	100
1/2MSR+glyphosate 0.05	10	0	0	10	9	90
1/2MSR+glyphosate 0.1	10	0	0	10	9	90
1/2MSR+glyphosate 0.15	10	0	0	10	8	80
1/2MSR+glyphosate 2	10	0	0	10	9	90
1/2MSR+glyphosate 4	10	0	0	10	9	90

Supplementary Table 4. Effects of different concentration glyphosate on seed germination.

Medium+glyphosate (mM/L)	Non-transgenic seeds			Transgenic seeds		
	No.of seeds inoculated	No.of germinated seeds	Ratio (%)	No.of seeds inoculated	No.of germinated seeds	Ratio (%)
1/2MSR	10	10	100	10	10	100
1/2MSR+glyphosate 0.01	10	10	100	10	10	100
1/2MSR+glyphosate 0.05	10	0	0	10	9	90
1/2MSR+glyphosate 0.2	10	0	0	10	10	100
1/2MSR+glyphosate 0.4	10	0	0	10	10	100
1/2MSR+glyphosate 0.6	10	0	0	10	9	90

Supplementary Table 5. The original data of Table 1

Vector	Selective Agent	No.of calli inoculated	No. of Resistant calli	Selection efficiency (%)	No.of calli for regeneration	No.of calli regenerating shoots	Regeneration efficiency (%)	No. of shots for rooting	No .of rooted plants	Rooting efficiency (%)	Positive transgene events by PCR (%)	Final transformation efficiency (%)
pCEi EPSPS	3mM glyphosate	158	101	63.92	27	26	96.3	18	14	77.78	100	47.88
		112	62	55.36	42	36	85.71	36	30	83.33	100	39.54
		160	74	46.25	48	46	95.83	32	28	87.5	100	38.78
	5mM glyphosate	212	113	53.3	57	52	91.23	32	29	90.63	100	44.07
		96	75	78.13	33	30	90.91	30	30	100	100	71.02
		288	127	44.1	48	46	95.83	35	32	91.43	100	38.64
pCP4-EPSPS	3mM glyphosate	128	100	78.13	38	30	78.95	26	22	84.62	100	52.19
		260	156	60	24	20	83.33	20	19	95	100	47.5
		160	70	43.75	36	30	83.33	30	30	100	100	36.46

	5mM glyphosate	224	158	70.54	51	45	88.24	39	33	84.62	100	52.66
		194	85	43.81	42	30	71.43	22	22	100	100	31.3
		220	140	63.64	54	48	88.89	35	34	97.14	100	54.95
pC1300	50mg/L hygromycin	204	105	51.47	46	40	86.96	25	18	72	85	32.23
		225	104	46.22	50	47	94	36	16	44.44	90	19.31
		486	220	45.27	66	57	86.36	43	35	81.4	85	31.82
